# Low-Temperature CVD Graphene Nanostructures on Cu and Their Corrosion Properties

**DOI:** 10.3390/ma11101989

**Published:** 2018-10-15

**Authors:** Wei-Hao Huang, Cheng-Hsuan Lin, Ben-Son Lin, Chia-Liang Sun

**Affiliations:** 1 Department of Chemical and Materials Engineering, Chang Gung University, Taoyuan 33302, Taiwan; clockp751681@yahoo.com.tw (W.-H.H.); ilovetaihsi@gmail.com (C.-H.L.); r93527066@ntu.edu.tw (B.-S.L.); 2 Department of Neurosurgery, Linkou Chang Gung Memorial Hospital, Taoyuan 33305, Taiwan

**Keywords:** low temperature, chemical vapor deposition, graphene films, copper, corrosion

## Abstract

Chemical vapor deposition (CVD) graphene is reported to effectively prevent the penetration of outer factors and insulate the underneath metals, hence achieving an anticorrosion purpose. However, there is little knowledge about their characteristics and corresponding corrosion properties, especially for those prepared under different parameters at low temperatures. Using electron cyclotron resonance chemical vapor deposition (ECR-CVD), we can successfully prepare graphene nanostructures on copper (Cu) at temperatures lower than 600 °C. Scanning electron microscopy (SEM), X-ray diffraction (XRD), Raman spectroscopy, and potentiodynamic polarization measurements were used to characterize these samples. In simulated seawater, i.e., 3.5 wt.% sodium chloride (NaCl) solution, the corrosion current density of one graphene-coated Cu fabricated at 400 °C can be 1.16 × 10^−5^ A/cm^2^, which is one order of magnitude lower than that of pure Cu. Moreover, the existence of tall graphene nanowalls was found not to be beneficial to the protection as a consequence of their layered orientation. These correlations among the morphology, structure, and corrosion properties of graphene nanostructures were investigated in this study. Therefore, the enhanced corrosion resistance in selected cases suggests that the low-temperature CVD graphene under appropriate conditions would be able to protect metal substrates against corrosion.

## 1. Introduction

Graphene has received numerous attentions since it was first detached from graphite through mechanical exfoliation and then used in a variety of applications [1,2,3,4,5,6,7,8,9,10,11,12,13,14,15]. Among all of the developed processes, chemical vapor deposition (CVD) is always viewed as an effective and powerful method for fabricating large-area and high-quality graphene. T J. Pang et al. discussed the CVD growth of one-dimensional (1D) and two-dimensional (2D) sp^2^ carbon nanomaterials, i.e., basically carbon nanotube and graphene, in 2015 [16]. Their growth mechanisms and dynamics were compared. Hofmann et al. reported on the chemical vapor deposition (CVD)-enabled graphene manufacture and technology in 2014 [17]. It mentioned the pressure and materials gap, the microstructure of CVD graphene films, and the catalyst−carbon phase diagram. Naghdi et al. reviewed the catalytic, catalyst-free, roll-to-roll, and low-temperature CVD growth of graphene in 2018 [18]. The role of the catalyst, carbon precursors, plasma assistance, catalyst-free, and roll-to-roll production all suggest that the research is still a long way from an economical and feasible approach for reducing the growth temperature. Chen et al. showed the scalable CVD growth of three-dimensional (3D) graphene materials in 2018 [19]. Graphene foams, graphene shells, hierarchical graphene, and their energy-related applications were presented. It is worthwhile to mention that few-layer epitaxial graphene nanowall arrays were able to be produced by microwave plasma-enhanced CVD with the help of thin SiC wall-like scaffolds [20].

On the other hand, graphene as an oxidation and corrosion resistive coating is of great interests recently [21,22,23,24,25,26,27,28,29,30]. For the oxidation resistance, Chen et al. showed this property of graphene-coated copper (Cu) and Cu/nickel (Ni) alloy against annealing at 200 °C in air [23]. Kalita et al. obtained graphene as an effective oxidation resistive barrier through the surface wave plasma CVD for against annealing at 150 °C in air [24]. Singh Raman et al. showed that graphene coating on Cu can increase the resistance to electrochemical degradation in 0.1 M of NaCl solution [25]. Prasai et al. reported on the corrosion-inhibiting graphene coating in Na_2_SO_4_ solution [26]. Hsieh et al. achieved the complete corrosion inhibition through graphene defect passivation using atomic layer deposition (ALD) [27]. Miskovic-Stankovic et al. indicated that graphene coating on Cu showed better protective properties than that on Al in 0.1 M of NaCl solution [28]. In contrast, Dong et al. claimed that the graphene film did not effectively protect Cu in the prolonged exposure to 3.5 wt.% NaCl solution [29]. Furthermore, Qi et al. synthesized the polymethylmethacrylate (PMMA)-grafted graphene oxide coating, which can prevent the corrosion of Cu in a 3.5 wt.% NaCl solution [30]. However, there is little information available on low-temperature chemical vapor deposition (LTCVD) graphene nanostructures and their electrochemical anticorrosion properties. Nevertheless, the high process temperatures may induce the phase transformation of various metal substrates and generate unnecessary changes in their mechanical as well as other properties, even only within a short time. On the other hand, to the best of our knowledge, there is no such study, and it is very difficult to achieve good coverage and high-quality graphene through low-temperature CVD with improved anticorrosion properties at the same time. Therefore, in this study, we prepared the CVD graphene-coated Cu substrates at and below 600 °C, and further investigated their electrochemical properties in a 3.5 wt.% NaCl solution. It is hoped that the information from this study would be useful for the development and design of novel anticorrosion coatings based on 2D materials. 

## 2. Materials and Methods

**Scheme 1:**
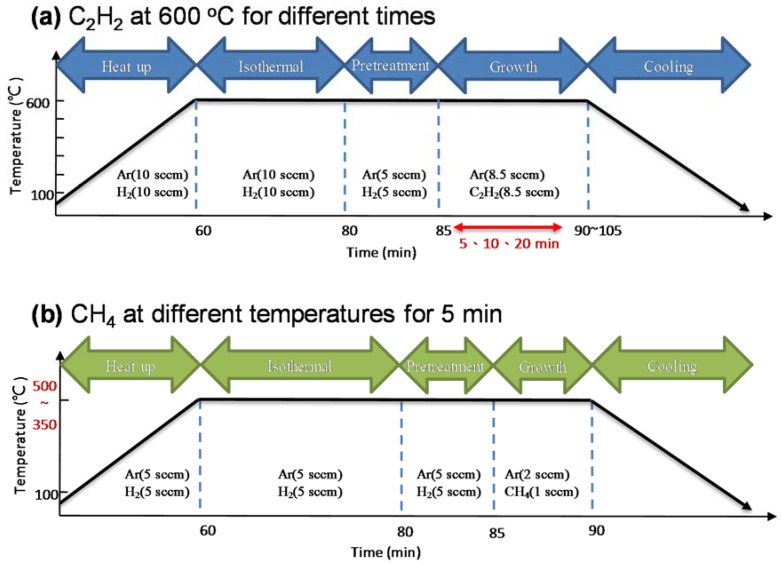
Multi-sources electron cyclotron resonance chemical vapor deposition system (MECR-CVD) (C_2_H_2_) and electron cyclotron resonance chemical vapor deposition system (ECR-CVD) (CH_4_) for graphene nanostructures under different conditions.

Cu foils (30 cm × 150 cm × 0.00254 cm) were obtained from Alfa Aesar (Lancashire, UK). Ag paste (OP-915, Double-O Technology Co., Ltd. Taipei, Taiwan) and pipe sealant (Loctite 5331, Henkel AG & Company, KGaA, Dusseldorf, Germany) were purchased for electrochemical measurements. A multi-sources electron cyclotron resonance chemical vapor deposition system (MECR-CVD, Industrial Technology Research Institute (ITRI), Hsinchu, Taiwan) was used for growing graphene films through flowing C_2_H_2_ at 600 °C for different times. On the other hand, electron cyclotron resonance chemical vapor deposition system (ECR-CVD, Industrial Technology Research Institute (ITRI), Hsinchu, Taiwan) was used for growing graphene films through flowing CH_4_ at different temperatures for 5 min. For film growth, the Cu foil (area: 2 cm × 2 cm) was used at the microwave power of 1200 W. The detailed rest process conditions and parameters were listed in Scheme 1. Since the MECR-CVD chamber is bigger than that of the ECR-CVD, we used a higher flow rate of precursor gases. After the pretreatment process, we used a gate valve to control the process pressures.

For material analyses, a field-emission scanning electron microscope (JEOL, JSM-7500F, Tokyo, Japan) and an X-ray diffractometer (Bruker, D2 Phaser, Billerica, MA, United States) were adopted. In addition, the graphene films on the Cu substrate were checked by a micro-Raman spectrometer (RAMaker Protrustech Co., Ltd. Tainan, Taiwan). Potentiodynamic polarization measurements were carried out by using a CHI potentiostat system (CHI 7032D, CH Instruments, Inc., Austin, TX, USA) in a typical three-electrode setup with an Ag/AgCl reference electrode and a Pt wire counter electrode. For preparing the working electrode, the Cu wire was mounted on the backside of Cu substrates through silver paste, while the backside and surrounding sides were protected by pipe sealant. The drying conditions of the silver paste and pipe sealant are 80 °C for 3.5 h and 40 °C for 30 min, respectively. The electrolyte is 3.5 wt.% NaCl solution, and the sweeping potential range is between −0.8 and 0.6 V at the scan rate of 10 mV/s. All of the solutions were prepared with deionized water with a resistivity of more than 18 megohm/cm. We used CorrView software (version 2.6) to do data fitting in the Tafel plot in order to get the corrosion potential.

## 3. Results and Discussion

**Figure 1:**
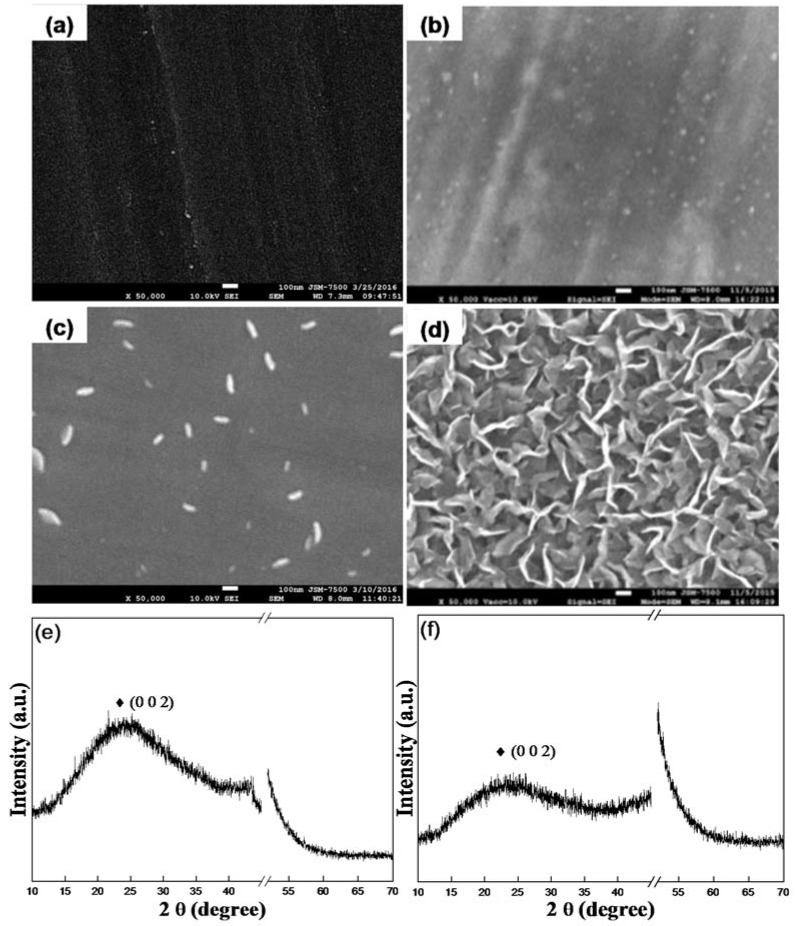
Scanning electron microscopy (SEM) pictures of (**a**) copper (Cu); (**b**) chemical vapor deposition (CVD) graphene (C_2_H_2_, 600 °C, 5 min) on Cu; (**c**) CVD graphene (CH_4_, 350 °C, 5 min) on Cu; (**d**) CVD graphene (CH_4_, 500 °C, 5 min) on Cu before electrochemical corrosion experiments. X-ray diffraction (XRD) patterns of (**e**) CVD graphene (C_2_H_2_, 600 °C, 5 min) on Cu; (**f**) CVD graphene (CH_4_, 350 °C, 5 min) on Cu.

Figure 1 shows the Scanning electron microscopy (SEM) pictures before the electrochemical corrosion experiments. Before the CVD growth, the Cu foil surface is relatively dark and smooth. After the CVD growth, the foil surface becomes bright and rough, with the existence of either graphene films or nanowalls. Figure 1b–d illustrates the surface morphologies with a variety of growth atmospheres, temperatures, and conditions. In Figure 1b, through the growth at 600 °C for 5 min with C_2_H_2_ flow, the surface texture seems not that clear, with few relatively large spots on gray surface. Figure 1c,d is picture through the growth at 350 °C and 500 °C for 5 min with CH_4_ flow, respectively. In the beginning, some rectangular stereostructures appear randomly at 350 °C, and gradually become larger through increasing temperatures, finally form the graphene nanowalls at 500 °C. In the later stages, even rose-like or flower-like structures start to stack and form big spheres under the influence of growth conditions. In general, graphene films grow for the most part in width, and not in height. While the film is thin, this coating appears flat. With a further increase in the amount of material in the film, it gets pinched and forms a texture. Figure 1e,f is the X-ray diffraction (XRD) patterns for samples with a variety of growth atmospheres, temperatures, and conditions. The two theta angles were from 10 to 70 degrees, and we ignored between 45–52 degrees, so that the strong signals from the Cu foil substrates did not interfere. It was found that there is a small and broad peak ranging from 20 to 30 degrees in both patterns. It could correspond to the graphite (002) peak located at 26.53 degrees (PDF card 00-056-0159). The relatively smaller full width at half maximum (FWHM) of Figure 1e than that of Figure 1f indicates that a larger grain along the c axis and better crystallinity for the growth at 600 °C with C_2_H_2_ flow. These large grain and good crystallinity would be attributed to the high growth temperature and better horizontal coverage. On the other hand, because the carbon ratio of C_2_H_2_ is higher than CH_4_, the C_2_H_2_ precursor could get more C_2_ radicals, which might be helpful for film deposition.

**Figure 2:**
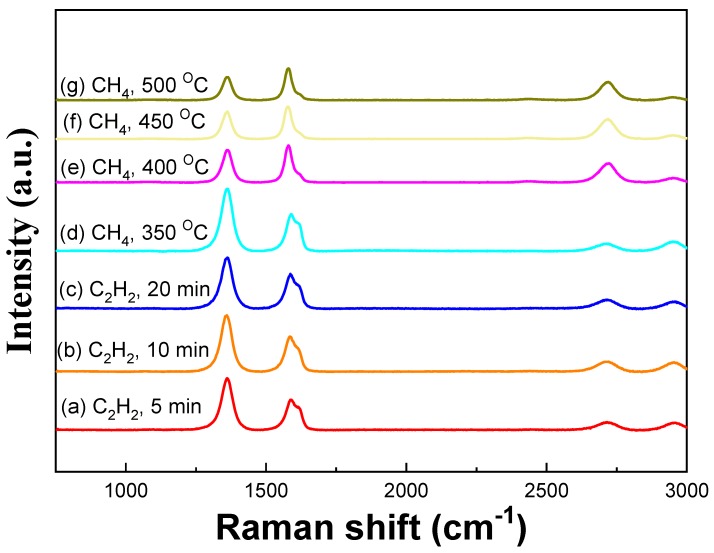
Raman spectra of chemical vapor deposition (CVD) graphene using C_2_H_2_ as carbon source at 600 °C for (**a**) 5 min, (**b**) 10 min, and (**c**) 20 min on Cu; Raman spectra of CVD graphene using CH_4_ as carbon source at (**d**) 350 °C, (**e**) 400 °C, (**f**) 450 °C, and (**g**) 500 °C for 5 min on Cu.

Figure 2a–g is the Raman spectra for samples with a variety of growth atmospheres, temperatures, and conditions with the laser excitation wavelength of 473 nm. There are Raman peaks at 1350 cm^−1^ (D band), 1590 cm^−1^ (G band), 1620 cm^−1^ (D′ band), 2690 cm^−1^ (2D band), and 2950 cm^−1^ (D + G band). These peaks concur the existence of graphene films and nanowalls on Cu foil substrates that are observed in the above SEM and XRD analyses. First, the small 2D peaks imply multilayer graphene structure for all of the cases instead of a few-layer or even a single-layer structure. For the Figure 2a–c with C_2_H_2_ flow, the growth time is the parameter that we controlled at the same temperature of 600 °C. The decreasing I_D_/I_G_ ratios are 1.70, 1.60, and 1.47 as a result of the better crystallinity for long growth times. For the Figure 2d−g with CH_4_ flow, the growth temperatures are 350 °C, 400 °C, 450 °C, and 500 °C with the same time of 5 min. In Table 1, the decreasing I_D_/I_G_ ratios are 1.69, 0.87, 0.84 and 0.72 for the increasing growth temperatures. Here, the high G-band intensity at higher growth temperatures is due to the much superior sp^2^ crystallization of graphene nanostructures compared with that of 300 °C. Especially, there is a rapid transition from 350 °C to 400 °C, and the finally forming non-flat and porous graphene nanowalls at 500 °C.

**Figure 3:**
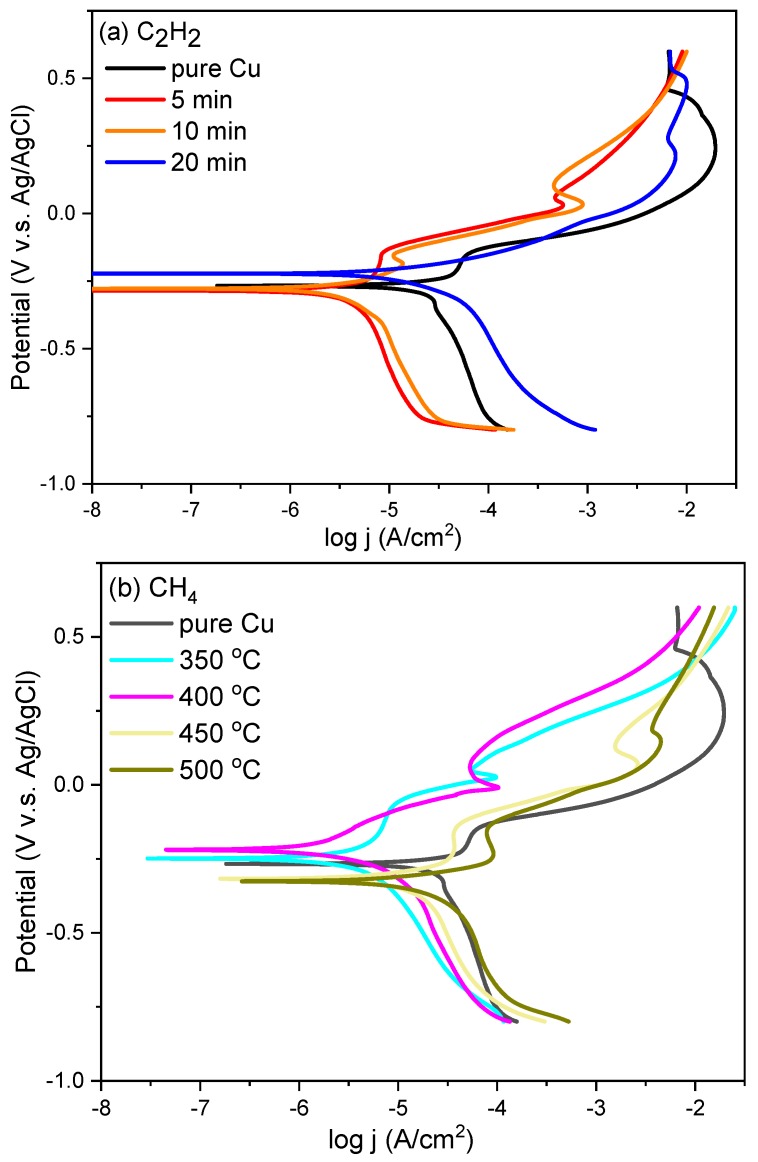
Potentiodynamic polarization curves of Cu and LTCVD graphene nanostructures grown on Cu using (**a**) C_2_H_2_ at 600 °C for different times and using (**b**) CH_4_ at different temperatures for 5 min for the first scan (electrolyte: 3.5 wt.% NaCl solution, scan rate: 10 mV/s).

**Figure 4:**
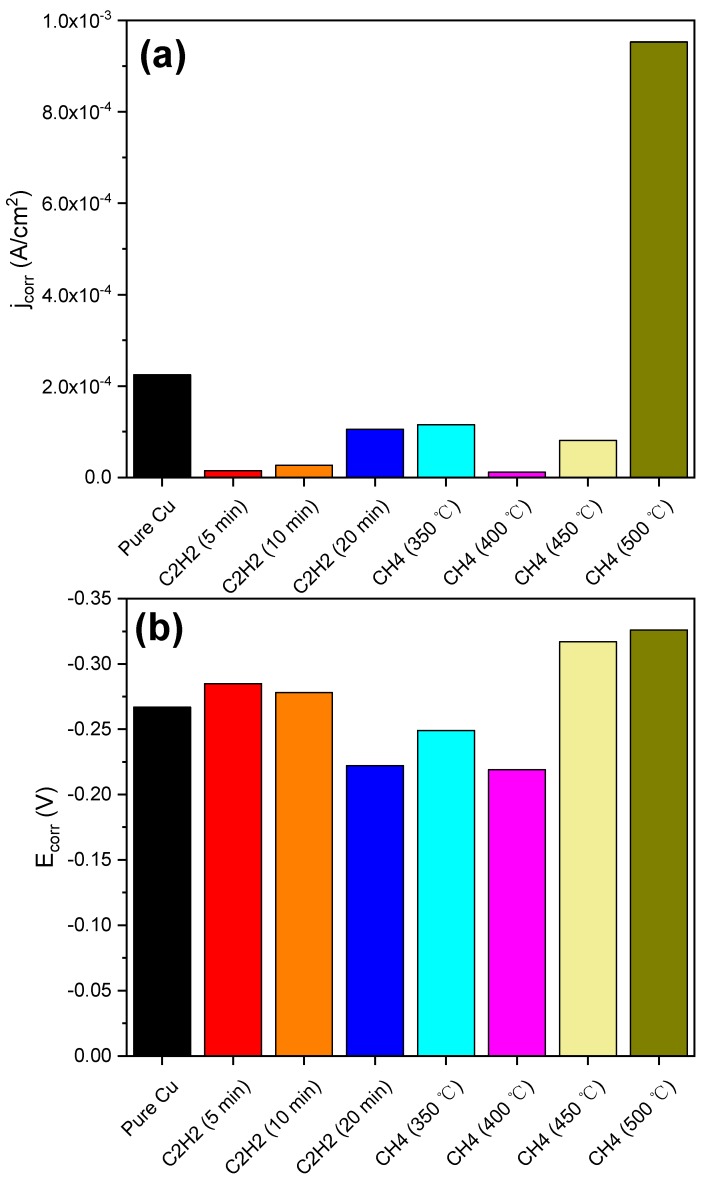
Summary of (**a**) j_corr_ and (**b**) E_corr_ of Cu and LTCVD graphene nanostructures grown on using different growth conditions (electrolyte: 3.5 wt.% NaCl solution, scan rate: 10 mV/s).

Figure 3 indicates the potentiodynamic polarization curves of the first scan for pure, CVD graphene (C_2_H_2_, 5 min)-coated, and CVD graphene (CH_4_, 350 °C)-coated Cu foils. The experiments were carried out ranging from −0.8 V to 0.6 V v.s. Ag/AgCl at the scan rate of 10 mV/s at room temperature. Originally, the corrosion potential for pure Cu foil is around −0.267 V. After adding good CVD graphene coatings, the corrosion potentials are shifted toward −0.2 V with several tens mV with relatively low corrosion activities. It depicts that the suitable CVD graphene coatings are able to improve the anticorrosion property under the appropriate growth conditions. Figure 4a,b is the summary of electrochemical corrosion current densities (j_corr_) and corrosion potential (E_corr_) for the first scan. In addition, the scan numbers were raised 30 times in order to investigate the long-term stability. The corrosion current densities (j_corr_) and corrosion potentials (E_corr_) for CVD graphene (C_2_H_2_)-coated Cu foils at different growth times and pure Cu foil were displayed in the left-hand side of Figure 4a,b, respectively. The j_corr_ of all of the CVD graphene (C_2_H_2_)-coated ones are lower than that of pure Cu foil. It is worthwhile to mention that the E_corr_ for graphene (C_2_H_2_, 20 min) is closer to the positive side with lower j_corr_, although it is relatively higher than those with shorter growth times. The right-hand side of Figure 4a,b illustrated the j_corr_ and E_corr_ for CVD graphene (CH_4_)-coated Cu foils at different growth temperatures and pure Cu foil, respectively. Despite the curve for 500 °C, all of the remaining j_corr_ are lower than that of pure Cu foil. However, the E_corr_ for graphene (CH_4_, 450 °C and 500 °C)-coated Cu foils are closed to the more negative potentials. This indicates that the appearance of vertical structures of graphene nanowalls might be fatal to the protection as a consequence of the fast diffusion channel against the horizontal surface. Among all of the growth conditions, the champion film is CVD graphene (CH_4_, 400 °C, 5 min), and its j_corr_ and E_corr_ are 1.16 × 10^−5^ A/cm^2^ and −0.219 V. Therefore, it is claimed that the protection graphene nanostructure can be successfully achieved at a relatively low temperature of 400 °C for 5 min in our system. 

**Figure 5:**
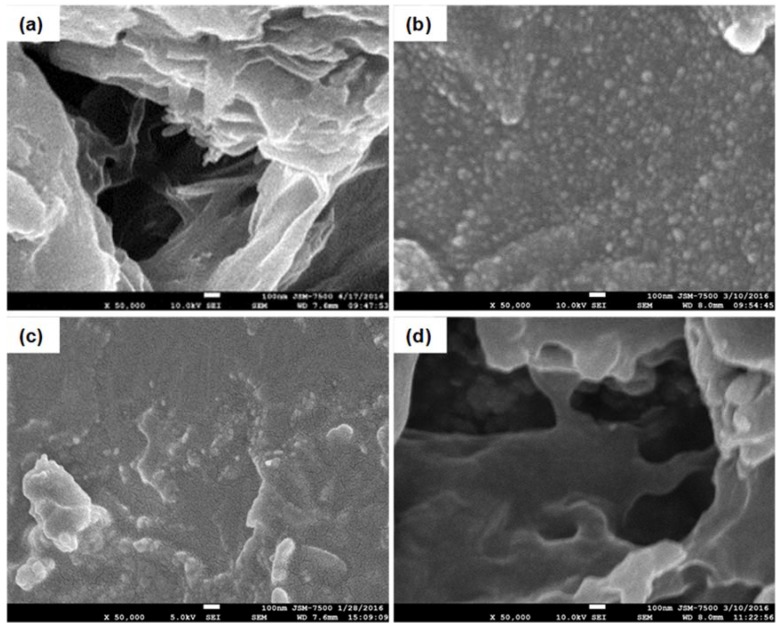
SEM pictures of (**a**) Cu; (**b**) CVD graphene (C_2_H_2_, 600 °C, 5 min) on Cu; (**c**) CVD graphene (CH_4_, 350 °C, 5 min) on Cu; (**d**) CVD graphene (CH_4_, 500 °C, 5 min) on Cu after the electrochemical corrosion experiments with a total of 30 scans.

Figure 5a–d show the SEM pictures after the electrochemical corrosion experiments. After washing surfaces using the deionized water, all of the surfaces are not as flat as those before the electrochemical measurements. As shown in Figure 5a,d, some micrometer-size holes start to appear on pure and CVD graphene (CH_4_, 500 °C)-coated Cu foils. Hence, basically the porous and high graphene nanowalls do not help the protection against corrosion in terms of morphology. In contrast, the surface with a low CVD growth temperature of 350 °C seems better. Figure 5b demonstrates the flattest surface with CVD graphene (C_2_H_2_, 5 min) coating. This flatness is correlated with the relatively low j_corr_ and E_corr_ on the more positive potential side. It indicates that the intermediate temperature CVD graphene with C_2_H_2_ as the carbon source can form the complete protection layer in a short growth time of five minutes, even suffering from multiple scans. Still, the corrosion will cause the surface oxidation confirmed by comparing the energy-dispersive x-ray spectroscopy (EDS) spectra (Figure S1 in Supplementary Materials). Similar trends can be found for graphene (CH_4_, 350 °C) and graphene (CH_4_, 400 °C) coatings. However, after 10 scans, the E_corr_ of pure Cu will shift to around −0.16 V, which could be a consequence of forming a passivation layer and thus be higher than those of all of the graphene-coated ones.

## 4. Conclusions

In conclusion, we have successfully demonstrated LTCVD graphene nanostructures with promising anticorrosion characteristics. The low j_corr_ and E_corr_ closed to the more positive side of graphene-coated Cu can be achieved simultaneously for selected and optimal cases. More importantly, the lowest j_corr_ of graphene (CH_4_, 400 °C)-coated Cu is one order of magnitude lower than that of the pure Cu. Meanwhile, its E_corr_ of −0.219 V is 52 mV more positive than that of unmodified Cu. In contrast, the cases located outside the appropriate process window may suffer from non-uniform coverage, poor crystallinity, or porous nanowall structures, which also provide vertical diffusion pathway into the substrates. Our study suggests that the appropriate process and associated structures are essential to the development of graphene-based anticorrosion coatings through LTCVD.

## Figures and Tables

**Table 1:** Characteristics of Raman spectra for low-temperature chemical vapor deposition (LTCVD) graphene films using different conditions.

Sample	I_D_/I_G_	I_2D_/I_G_
C_2_H_2_, 5 min (600 °C)	1.70	0.24
C_2_H_2_, 10 min (600 °C)	1.60	0.28
C_2_H_2_, 20 min (600 °C)	1.47	0.25
CH_4_, 350 °C (5 min)	1.69	0.19
CH_4_, 400 °C (5 min)	0.87	0.51
CH_4_, 450 °C (5 min)	0.84	0.61
CH_4_, 500 °C (5 min)	0.72	0.51

## Supplementary Materials

The following are available online at http://www.mdpi.com/1996-1944/11/10/1989/s1, Figure S1: EDS spectra of CVD graphene (C_2_H_2_, 600 °C, 5 min) coatings before and after electrochemical corrosion experiments. Time-dependent J_corr_ and E_corr_ in the samples, Figure S2: Time-dependent J_corr_ and E_corr_ in these samples.
