# Supplementary material for: Low-Temperature CVD Graphene Nanostructures on Cu and Their Corrosion Properties
Source: Materials (Basel). 2018 Oct 15;11(10):1989. doi: 10.3390/ma11101989 (PMC6213400; doi:10.3390/ma11101989)
Supplement: Supplementary file 1 [file materials-11-01989-s001.pdf]

materials

MDPI

Supplementary

Low-Temperature CVD Graphene Nanostructures on Cu and their Corrosion Properties

Wei-Hao Huang [1], Cheng-Hsuan Lin [1], Ben-Son Lin [1] and Chia-Liang Sun [1,2,*]

[1] Department of Chemical and Materials Engineering, Chang Gung University, Taoyuan 33302, Taiwan; clockp751681@yahoo.com.tw (W.-H.H.); ilovetaihsi@gmail.com (C.-H.L.); r93527066@ntu.edu.tw (B.-S.L.)
[2] Department of Neurosurgery, Linkou Chang Gung Memorial Hospital, Taoyuan 33305, Taiwan
* Correspondence: clsun@mail.cgu.edu.tw; Tel.: +886-3-2118800, Ext. 5379

Received: 20 August 2018; Accepted: 2 October 2018; Published: 15 October 2018

(a)

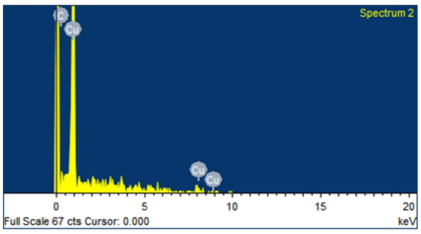

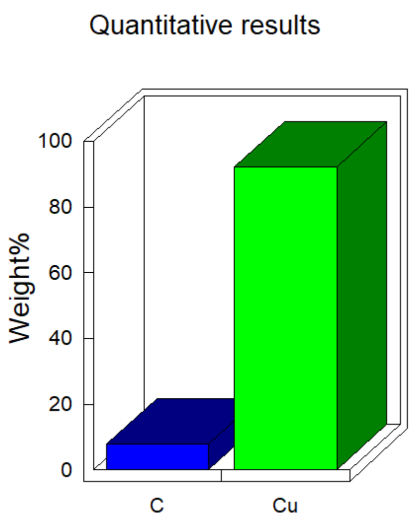

Element	Weight %	Atomic %
C K	7.79	30.89
Cu L	92.21	69.11
Total	100.00	100.00

(b)

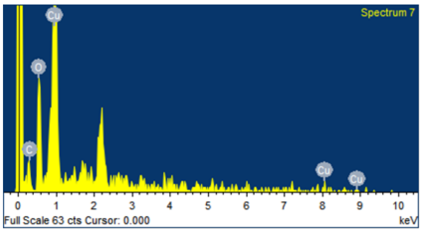

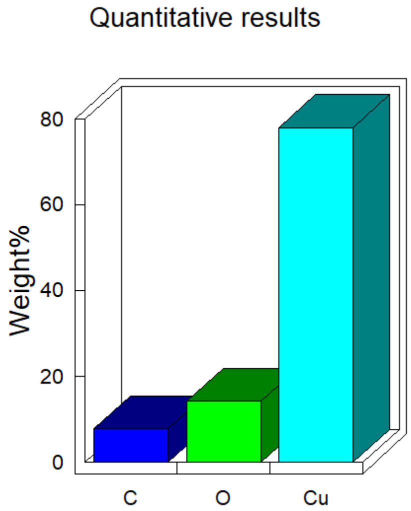

Element	Weight %	Atomic %
C K	7.79	23.45
O K	14.24	32.18
Cu L	77.97	44.38
Total	100.00	100.00

Figure S1. Energy-dispersive x-ray spectroscopy (EDS) spectra of CVD graphene (C_2H_2, 600 °C, 5 min) coatings on Cu (**a**) before and (**b**) after the electrochemical corrosion experiments with a total of 30 scans.

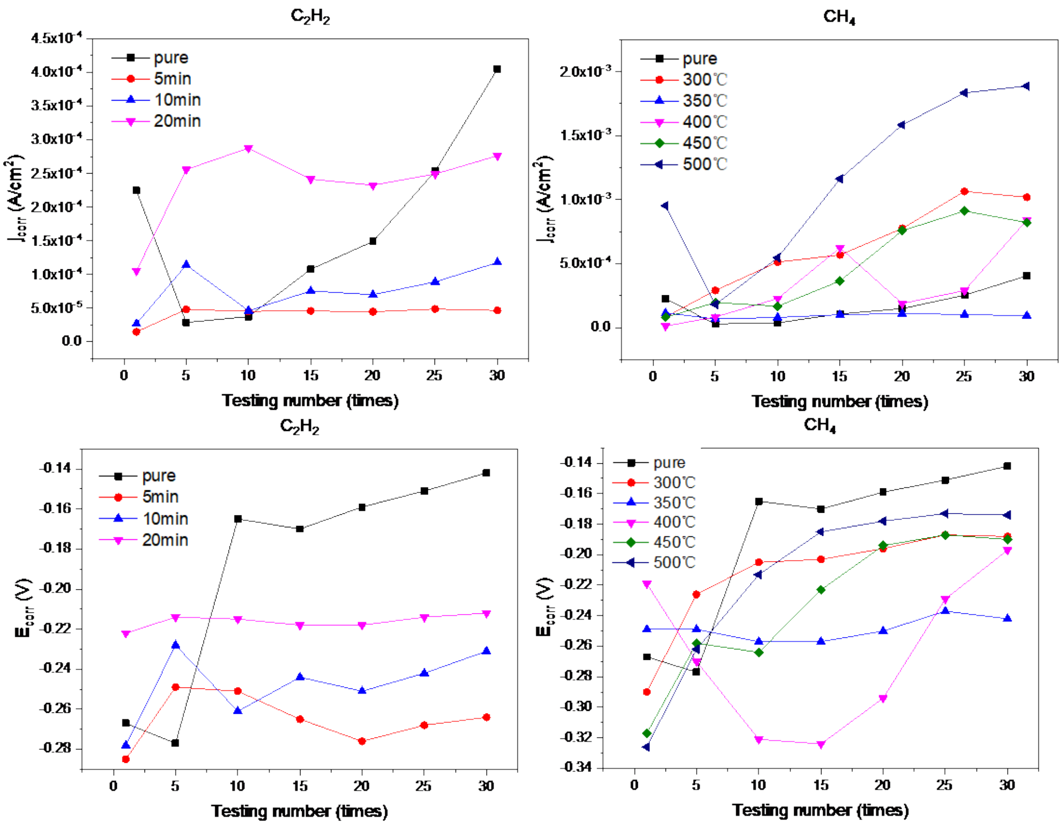

Figure S2. Time-dependent Jcorr and Ecorr in these samples.

© 2018 by the authors. Submitted for possible open access publication under the terms and conditions of the Creative Commons Attribution (CC BY) license (http://creativecommons.org/licenses/by/4.0/).
